# Dioscin elicits anti‐tumour immunity by inhibiting macrophage M2 polarization via JNK and STAT3 pathways in lung cancer

**DOI:** 10.1111/jcmm.15563

**Published:** 2020-07-02

**Authors:** Luyun Cui, Guangdie Yang, Jiani Ye, Yinan Yao, Guohua Lu, Junjun Chen, Liangjie Fang, Shan Lu, Jianying Zhou

**Affiliations:** ^1^ Department of Respiratory Medicine The First Affiliated Hospital College of Medicine Zhejiang University Hangzhou China

**Keywords:** anti‐tumour, dioscin, lung cancer, macrophages, polarization

## Abstract

Tumour‐associated macrophage (TAM) is an important component in tumour microenvironment. Generally, TAM exhibits the function of M2‐like macrophage, which was closely related to angiogenesis and tumour progression. Dioscin, a natural steroidal saponin, has shown its powerful anti‐tumour activity recently. However, the mechanism of dioscin involved in immune regulation is still obscure. Here, we observed dioscin induced macrophage M2‐to‐M1 phenotype transition in vitro and inhibited IL‐10 secretion. Meanwhile, the phagocytosis of macrophages was enhanced. In subcutaneous lung tumour models, dioscin inhibited the augmentation of M2 macrophage populations. Furthermore, dioscin down‐regulated STAT3 and JNK signalling pathways in macrophages in vitro. In BMDMs, activating JNK and inhibiting STAT3 induce macrophages to M1 polarization while inhibiting JNK and activating STAT3 to M2 polarization. Additionally, condition mediums from dioscin‐pre‐treated macrophages inhibited the migration of 3LL cells and the tube‐formation capacity of HUVECs. What's more, dioscin‐mediated macrophage polarization inhibited the in vivo metastasis of 3LL cells. In conclusion, dioscin may act as a new anti‐tumour agent by inhibiting TAMs via JNK and STAT3 pathways in lung cancer.

## 1 INTRODUCTION

Tumour microenvironment, consisting of tumour cells and surrounding non‐tumour components, is closely related to tumour progression and becomes a therapeutic target.^1^, ^2^ Immune cells in tumour microenvironment could be re‐educated and turn to facilitate tumour growth and metastasis.^3^ Tumour‐associated macrophages (TAMs) are typical of these, which have two types: M1‐like TAMs and M2‐like TAMs. M1‐like TAMs, playing a tumour suppression role, express some markers such as CD86, inducible nitric oxide synthase (NOS2), IL‐6, IL‐12, and IL‐23.^4^, ^5^, ^6^, ^7^, ^8^ M2‐like TAMs, contrast to M1‐like TAMs, are regarded as to be immunosuppressive and pro‐tumorigenic. They generally exhibit CD206, CD209, CD163, arginase 1 (Arg‐1) and IL‐10.^5^, ^6^, ^7^, ^8^, ^9^, ^10^, ^11^, ^12^ Previous studies have elucidated TAMs mainly exhibit the function of M2‐like macrophages.^9^, ^12^, ^13^, ^14^, ^15^ More specifically, the proportion of M2‐like TAMs is approximately 70% in human non–small‐lung cancers (NSCLCs).^14^ Overexpressed negative immune regulatory molecules of M2‐like TAMs, such as Arg‐1, IL‐10, programmed cell death 1 ligand (PD‐L1) and cytotoxic T lymphocyte antigen 4 (CTLA‐4), inhibited the effect of CD4^+^ and CD8^+^ T cells to induce immunosuppressive microenvironment for tumour development.^16^, ^17^, ^18^, ^19^, ^20^ Many other factors also exist in the tumour microenvironment, such as platelet‐derived growth factor (PDGF), vascular endothelial growth factor (VEGF), matrix metallopeptidase (MMP) and CCL.^2^, ^9^, ^15^ And the regulator network of these factors leads to angiogenesis, proliferation of malignant cells, tumour invasion and metastasis.^9^, ^15^, ^21^ What's more, in NSCLCs, the high ratio of M1/M2 TAMs and M1 TAMs is positively associated with patients' survival while M2 TAM accumulation with poor outcome.^12^, ^14^, ^18^ Therefore, intervention of M2 polarization may become a promising new strategy for lung cancer treatment.^13^

Dioscin, a natural steroidal saponin, is extracted from the roots of dioscorea plants, such as dioscorea zingiberensis and dioscorea nipponica.^22^ During recent years, the anti‐tumour effect of dioscin has been reported progressively.^23^, ^24^, ^25^, ^26^, ^27^ In human lung cancer cells, dioscin could inhibit TGF‐β1‐mediated epithelial‐mesenchymal transition, induce cell apoptosis and suppress tumour invasion.^27^, ^28^ Interestingly, some studies detect dioscin has the potential effect to reverse drug resistance.^29^, ^30^, ^31^ However, there are few studies focused on the effects of dioscin in immune regulation. It has been confirmed dioscin could induce Raw264.7 cells to M1 polarization and then up‐regulate connexin 43 expression to inhibit melanoma progression.^10^ But whether the anti‐tumour influence of dioscin is related to the effect on macrophage polarization and the detail mechanism has yet to be determined.

In the current study, we try to explore the impact of dioscin on phenotypes and functions of macrophages. We utilized in vitro cell culture systems (BMDMs and Raw264.7 cells) to elucidate dioscin‐induced phenotype transition from M2 to M1 with the down‐regulation of STAT3 and JNK. Then, we constructed a subcutaneous lung cancer model to confirm the inhibition of dioscin on macrophage M2 polarization in vivo. What's more, the phagocytosis of BMDMs was enhanced with dioscin treatment. With condition medium treated, we discovered dioscin could inhibit the migration of 3LL cells and the tube‐formation capacity of HUVECs. And our lung metastases models in vivo indicated dioscin‐mediated macrophage polarization inhibited the metastasis of 3LL cells. In conclusion, our results suggested dioscin elicits anti‐tumour immunity by inhibiting macrophage M2 polarization through JNK and STAT3 pathways in lung cancer.

## 2 MATERIAL AND METHODS

### 2.1 Cell lines and reagents

Raw264.7 cells and Human Umbilical Vein Endothelial Cells (HUVECs) were obtained from the American Type Culture Collection (ATCC; Manassas, VA, USA). The cell line, 3LL, was a gift from Institute of Immunology, Zhejiang University School of Medicine. All cells were cultured in DMEM (NORTHEND, Hangzhou, China) with 10% foetal bovine serum (FBS, Gibco BRL Co., Ltd., Houston, TX, USA), 100 U/mL penicillin and 100 U/mL streptomycin (Solarbio, Beijing, China), at 37℃ in 5% CO_2_. Dioscin was purchased from Shanghai Tauto Biochemical Technology Co., Ltd (Shanghai, China). Stock solution of dioscin was made at 20 mmol/L concentration in dimethyl sulphoxide (DMSO) (Sangon Biotech, Shanghai, China) and stored at −20℃. The final concentration of DMSO for all treatments was less than 0.1%.

### 2.2 Isolation and culture of BMDMs

Male C57BL/6 mice (5‐6 weeks of age, SPF) were purchased from Shanghai SLAC Laboratory Animal Co., Ltd (Shanghai, China). To isolate bone marrow–derived macrophages (BMDMs), pelvic and femoral bones were dissected and bone marrow cells were expelled into PBS (NORTHEND, Hangzhou, China). Then, cells were passed through a 100‐μm cell strainer (BD Falcon, NY, USA) and resuspended in Red Blood Cell Lysis Buffer (Solarbio, Beijing, China) for 5 minutes to remove red blood cells. Finally, cells were centrifuged and cultured at 37℃ in 5% CO_2_ for 7 days in DMEM medium (100 U/mL penicillin and 100 U/mL streptomycin; 10% FBS) with 5 ng/mL murine recombinant M‐CSF (Peprotech, Rocky Hill, NJ, USA).

### 2.3 Cell cytotoxicity assay

Raw264.7 cells and BMDMs were seeded in 96‐well plates, and treated with control (0.1% DMSO) and different concentrations of dioscin for 24, 48 hours. After adding cell counting kit‐8 (Dojindo Laboratories, Tokyo, Japan) for 1‐ to 4‐hours incubation, the OD value was read by SpectraMax i3x Multi‐Mode Microplate Reader (Molecular Devices, San Francisco, CA, USA) at 450 nm.

### 2.4 Animal experiment

Male C57BL/6 mice (6‐8 weeks) were purchased from Shanghai SLAC Laboratory Animal Co., Ltd. 2 × 10^5^ suspended 3LL cells were injected into the right axilla of each mouse. After 4 days, the mice were randomized into 3 groups. Each group of mice was given with 0.5% sodium carboxymethylcellulose (Sangon Biotech, Shanghai, China) or dioscin (30 and 60 mg/kg/d) by gavage for 17 days. During the experiment, mice were sacrificed when the tumour diameter exceeded 2 cm. At the endpoint, blood, tumours and spleens were collected for further analysis.

Then, we established lung metastases mouse model: C57BL/6 mice were divided into 4 groups: control, dioscin treatment, clodronate liposome treatment and dioscin combined with clodronate liposome treatment (n = 4 per group). Mice were injected intravenously with 1 × 10^6^ 3LL cells in 0.1 mL PBS. Clodronate liposome or control liposomal (Liposoma, Amsterdam, Netherlands) was given to the mice by intraperitoneal injection at a dose of 100 μL/10 g daily for 3 days before tumour‐cell injection, followed by repeated injections of 50 μL/10 g every fourth day. Dioscin was given at 60 mg/kg by gavage 24 hours before tumour‐cell injection, and once a day thereafter for prolonged treatments.

We also established co‐inoculated lung metastasis mouse model: Raw264.7 cells were pre‐treated with dioscin for 48 hours. And Raw264.7 cells pre‐treated or not were co‐injected intravenous with 1 × 10^6^ 3LL cells in a ratio of 3:1 in 200 μL DMEM into C57/BL6 mice (n = 4 per group).

For analysis of pulmonary metastases, 21 days after injection of tumour cells, lungs were removed and fixed in formalin, cut in 3‐μm sections every 200 μm and stained for haematoxylin and eosin. Finally, the number of metastases was counted and assigned to the respective size category: small (diameter: <200 μm), medium (diameter: 200‐400 μm) or large (diameter: >400 μm).

The animal experiment was performed according to the Regulations for the Administration of Affairs Concerning Experimental Animals and was approved by the Experimental Animal Ethics Committee of the first Affiliated Hospital, College of Medicine, Zhejiang University.

### 2.5 Flow cytometry

BMDMs were pre‐treated and then stained in PBS with cell surface–specific antibodies for 30 mins at room temperature in the dark. After washing, the cells were analysed. In case of intracellular staining, cells were fixed with IC Fixation Buffer (eBioscience, San Diego, CA, USA) for 20‐60 minutes at room temperature in the dark and then wash cells twice with Permeabilization Buffer (eBioscience, San Diego, CA, USA). Next, cells were stained with fluorochrome conjugated antibodies for 30 minutes at room temperature in the dark. After washing, the cells were resuspended in PBS and analysed. As for detection of intracellular cytokines, BMDMs were treated with dioscin for 48 hours and then were stimulated by 100 ng/mL LPS (Sigma‐Aldrich, St. Louis, MO, USA) overnight. Next day, protein transport inhibitor cocktail (eBioscience, San Diego, CA, USA) was used to block secretion of cytokines. After stained with F4/80, BMDMs were stained with fluorochrome‐conjugated cytokines antibodies by intracellular staining. Finally, cells were analysed using a FACSVerse flow cytometer (BD Biosciences, New York, NY, USA). The staining antibodies used for flow cytometry are as follows: anti‐mouse F4/80‐Pacific Blue, CD86‐PE/CY7 and CD206‐PE were purchased from BioLegend (San Diego, CA, USA). Anti‐mouse CD209‐PE and NOS2‐APC were obtained from eBioscience (San Diego, CA, USA), while anti‐mouse IL‐10‐APC and IL‐12‐PE were obtained from BD Pharmingen (San Diego, CA, USA).

### 2.6 Quantitative real‐time PCR

Cells were extracted using RNA‐Quick Purification Kit according to the manufacturer's instructions (Yishan Biotech, Shanghai, China). The RNA concentration was measured using a NanoDrop 2000 (Thermo Scientific, Waltham, MA, USA) and then was converted into cDNA using a reverse transcription system (Takara, Shiga, Japan). Quantitative real‐time PCR was performed in a CFX96 Real‐Time PCR Detection System (Bio‐Rad) with SYBR Green Master Mix (Yeasen Biotech, Shanghai, China). The primer pairs were synthesized by Sangon Biotech Co., Ltd (Shanghai, China). Each experiment was performed in triplicate. Data are displayed as 2^‐ΔΔCt^ values, and GAPDH was used as an internal control. All primers used for quantitative real‐time PCR are as follows: GAPDH, (F) 5′‐AGGTCGGTGTGAACGGATTTG‐3′ and (R) 5′‐TGTAGACCATGTAGTTGAGGTCA‐3′; Arg1, (F) 5′‐CTCCAAGCCAAAGTCCTTAGAG‐3′ and (R) 5′‐AGGAGCTGTCATTAGGGACATC‐3′; CD206, (F) 5′‐CTCTGTTCAGCTATTGGACGC‐3′ and (R) 5′‐CGGAATTTCTGGGATTCAGCTTC‐3′; NOS2, (F) 5′‐GTTCTCAGCCCAACAATACAAGA‐3′ and (R) 5′‐GTGGACGGGTCGATGTCAC‐3′; IL‐6, (F) 5′‐TAGTCCTTCCTACCCCAATTTCC‐3′ and (R) 5′‐TTGGTC CTTAGCCACTCCTTC‐3′.

### 2.7 Western blot analysis

After the different treatment, cells were lysed in RIPA lysis buffer (Beyotime, Shanghai, China). Then, the protein was separated by SDS‐PAGE, transferred to PVDF membranes (Millipore, Bedford, MA, USA) and probed with the indicated primary antibodies for overnight at 4℃. After incubated with the corresponding secondary antibodies for 1 hour, the densities of bands were examined by ECL Chemiluminescence Kit HRP (FDbio, Hangzhou, China). Antibodies against ERK, p‐ERK (Thr202/Thy204), p38, p‐p38(Thr180/Tyr182) were purchased from Cell Signaling Technology (Danvers, MA, USA). Antibodies against JNK, p‐JNK(Thr183) and p‐STAT3(Tyr705) were purchased from Diag Biotechnology (Hangzhou, China). The antibody against STAT3 was obtained from Affinity Biosciences (Cincinnati, OH, USA). The antibody against GAPDH was obtained from Beyotime (Shanghai, China).

### 2.8 Phagocytosis assay

Phagocytosis is determined by the amount of fluorescence‐labelled latex beads (Sigma‐Aldrich, St. Louis, MO, USA) internalized by BMDMs. BMDMs were seeded in 6‐well plates and treated with dioscin for 48 hours. Then, BMDMs were incubated with fluorescent latex beads for 1‐2 hours. After washing, BMDMs were analysed by microscopy (Olympus, Tokyo, Japan) or were detached by trypsin and centrifuged in PBS for flow cytometry.

### 2.9 Inhibitors and activators

SP600125 (a JNK inhibitor), Stattic (a STAT3 inhibitor) and Anisomycin (a JNK activator) were purchased from Selleck Chemicals (Houston, TX, USA). Colivelin, a STAT3 activator, was purchased from Santa Cruz Biotechnology (Santa Cruz, CA, USA). BMDMs were treated with different inhibitors and activators alone or with dioscin, then were analysed by quantitative real‐time PCR and Western blot.

### 2.10 Preparation of conditioned medium

Raw264.7 cells and BMDMs were seeded in 6‐well plates with dioscin for 48 hours, then replaced with fresh serum‐free DMEM medium for another 24 hours and collected these media as conditioned medium. After centrifugation, supernatant was collected and stored at −20℃.

### 2.11 Transwell assay

3LL cells were starved for 12 hours and then cultured in the upper chamber (24‐well Transwell chambers, 8‐μm pore size, Corning, NY, USA) with serum‐free medium, while high‐serum medium (5% FBS) with condition medium was added to the lower chamber. After 24 hours of incubation at 37℃, cells were fixed with methanol and stained with 0.1% crystal violet for 30 minutes. Images were taken using microscope (Olympus, Tokyo, Japan).

### 2.12 Tube formation assay

96‐well plates were pre‐treated with 50 μL Matrigel (BD Bioscience, SFO, USA) per well and were placed at 37℃ for 1 hour. HUVECs were resuspended in serum‐free DMEM medium with condition medium and then cultured in 96‐well plate with Matrigel at 37℃ for 4‐6 hours. Images were taken using microscope (Olympus, Tokyo, Japan).

### 2.13 Statistical analysis

All data were presented as the means ± SD. The statistical significance was assessed by Student's t test using Prism 6.04 software (GraphPad Software Inc, San Diego, CA, USA). *P < 0.05, **P < 0.01, ***P < 0.001 are determined as significance.

## 3 RESULTS

### 3.1 Determination of BMDMs and detection of the non‐cytotoxic concentration of dioscin in Raw264.7 cells and BMDMs

**FIGURE 1:**
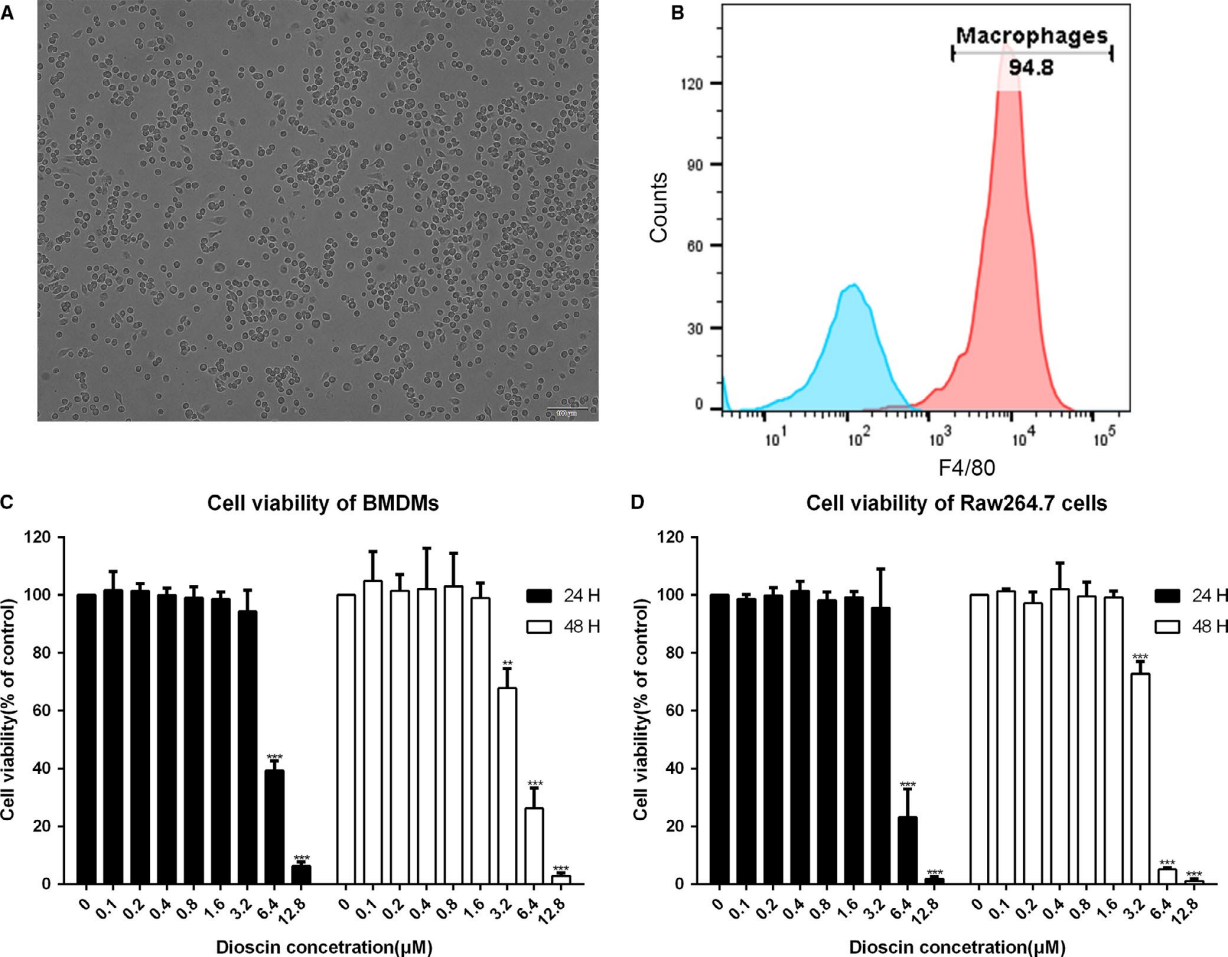
Determination of BMDMs and detection of the non‐cytotoxic concentration of dioscin in macrophages. A‐B, Image of BMDMs was obtained under a light microscope. Scale bars: 100 μm. The percentage of F4/80^+^ cells as obtained by flow cytometry. C‐D, Detection of the non‐cytotoxic dose of dioscin. BMDMs and Raw264.7 cells were incubated with dioscin at range from 0 to 12.8 μmol/L for 24 h and 48 h. Cell viability was determined by CCK‐8 assay and was performed in triplicate, thrice independently. The data were presented as mean ± SD. *P < 0.05, **P < 0.01 and ***P < 0.001 vs control

On the 7th day of culture, image of BMDMs was obtained under a light microscope (Figure 1A). And the per cent of F4/80^+^ cells by flow cytometry was over 90% (Figure 1B). We then evaluated the cytotoxicity of dioscin ranging from 0.1 to 12.8 μmol/L in Raw264.7 cells and BMDMs. Compared with the control treatment, dioscin began to inhibit the viability of RAW264.7 cells and BMDMs from the concentration of 3.2 μmol/L at 48 hours (Figure 1C,D). The half maximal inhibitory concentration (IC50) of RAW264.7 cells and BMDMs was 3.809 μmol/L and 4.319 μmol/L, respectively.

### 3.2 Dioscin‐induced macrophage phenotype transition from M2 to M1 in vitro

**FIGURE 2:**
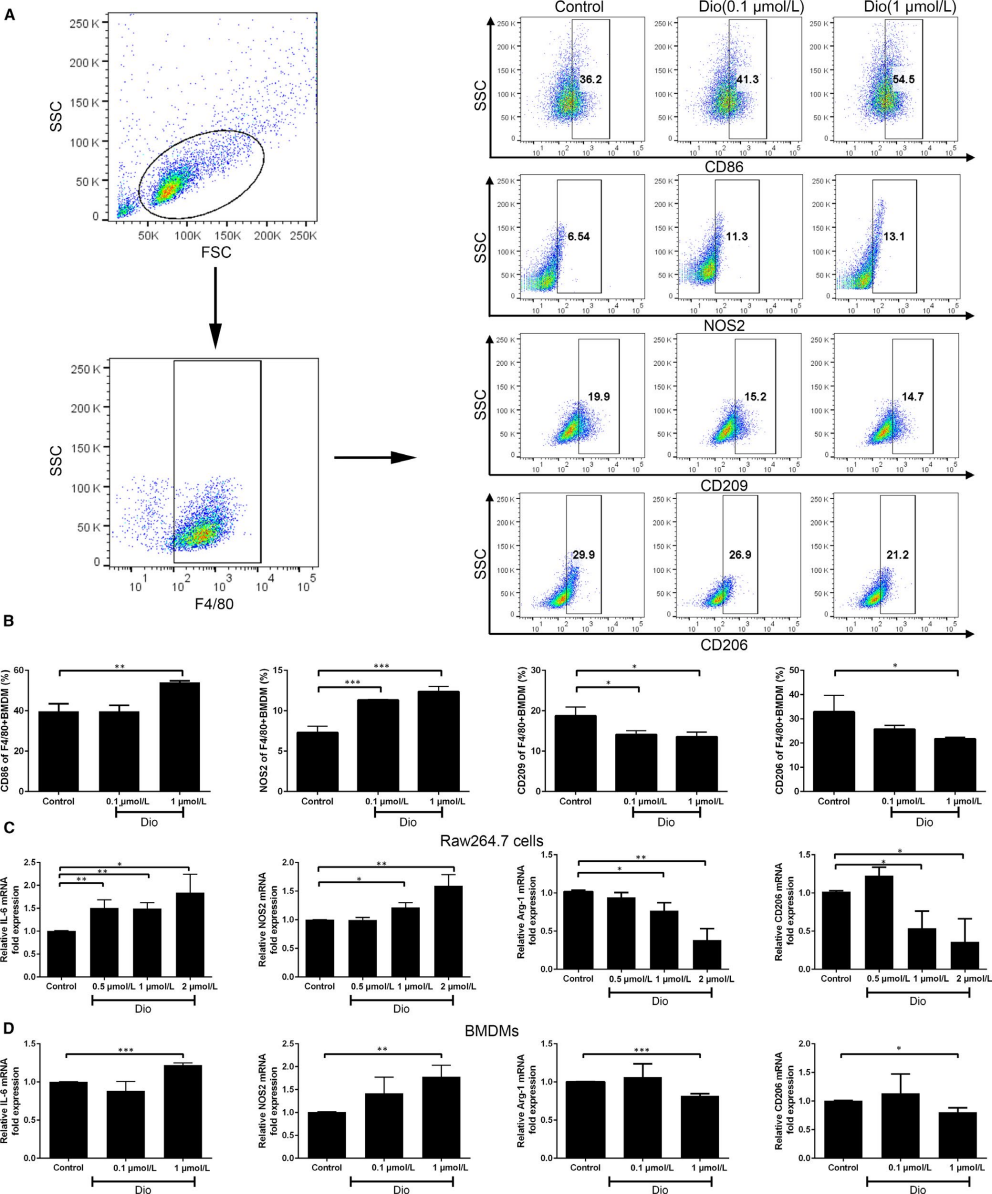
Dioscin induced macrophage phenotype transition from M2 to M1 in vitro. BMDMs and RAW264.7 cells were treated with dioscin for 48 h. A‐B, The numbers of F4/80^+^CD86^+^ (M1), F4/80^+^NOS2^+^ (M1), F4/80^+^CD206^+^ (M2) and F4/80^+^CD209^+^ (M2) cells among the total F4/80^+^ cells were quantified. C‐D, Relative mRNA expressions of the M1 genes IL‐6, NOS2 and the M2 genes Arg‐1, CD206 were measured by qPCR. The data were representatives of three independent experiments and presented as the mean ± SD. *P < 0.05, **P < 0.01 and ***P < 0.001 vs control

To study the effect of dioscin on BMDMs polarization, we used flow cytometry to analyse the percentage of M1 macrophages and M2 macrophages after dioscin treatment. M1 macrophages were defined as F4/80^+^CD86^+^ cells or F4/80^+^NOS2^+^ cells, while M2 macrophages were F4/80^+^CD206^+^ cells or F4/80^+^CD209^+^ cells. As shown in Figure 2, compared with the control group, both 0.1 μmol/L dioscin and 1 μmol/L dioscin significantly enhanced the expression of M1 phenotype biomarkers NOS2 in F4/80^+^cells (11.33 ± 0.06%, 12.37 ± 0.64% vs 7.31 ± 0.79%, respectively, both *P* < 0.001). And 1 μmol/L dioscin dramatically expanded the proportion of F4/80^+^CD86^+^ cells (54.03 ± 0.72% vs 39.70 ± 3.70%, *P < *0.01) (Figure 2A,B). Dioscin treatment (0.1 μmol/L and 1 μmol/L) also decreased the percentage of F4/80^+^CD209^+^ cells (14.10 ± 0.95% and 13.57 ± 1.15% vs 18.77 ± 2.14%, respectively, both *P* < 0.05) (Figure 2A,B). Simultaneously, the proportion of F4/80^+^CD206^+^cells were decreased with 1 μmol/L dioscin treatment from 32.90 ± 6.82% to 21.73 ± 0.68% (*P* < 0.05).

The M1 and M2 phenotypes were further confirmed by gene expression analysis in Raw264.7 cells and BMDMs. We measured the expression of M1 genes (IL‐6 and NOS2) and M2 genes (Arg‐1 and CD206) by qPCR. As shown in Figure 2C, dioscin‐treated groups showed significant up‐regulation of M1 and down‐regulation of M2 relevant gene expression in Raw264.7 cells. BMDMs treated with 1 μmol/L dioscin also had higher IL‐6 and NOS2 mRNA levels but lower Arg1 and CD206 mRNA levels (Figure 2D).

### 3.3 Dioscin restrained the augmentation of M2 macrophage populations in mice with lung cancer

**FIGURE 3:**
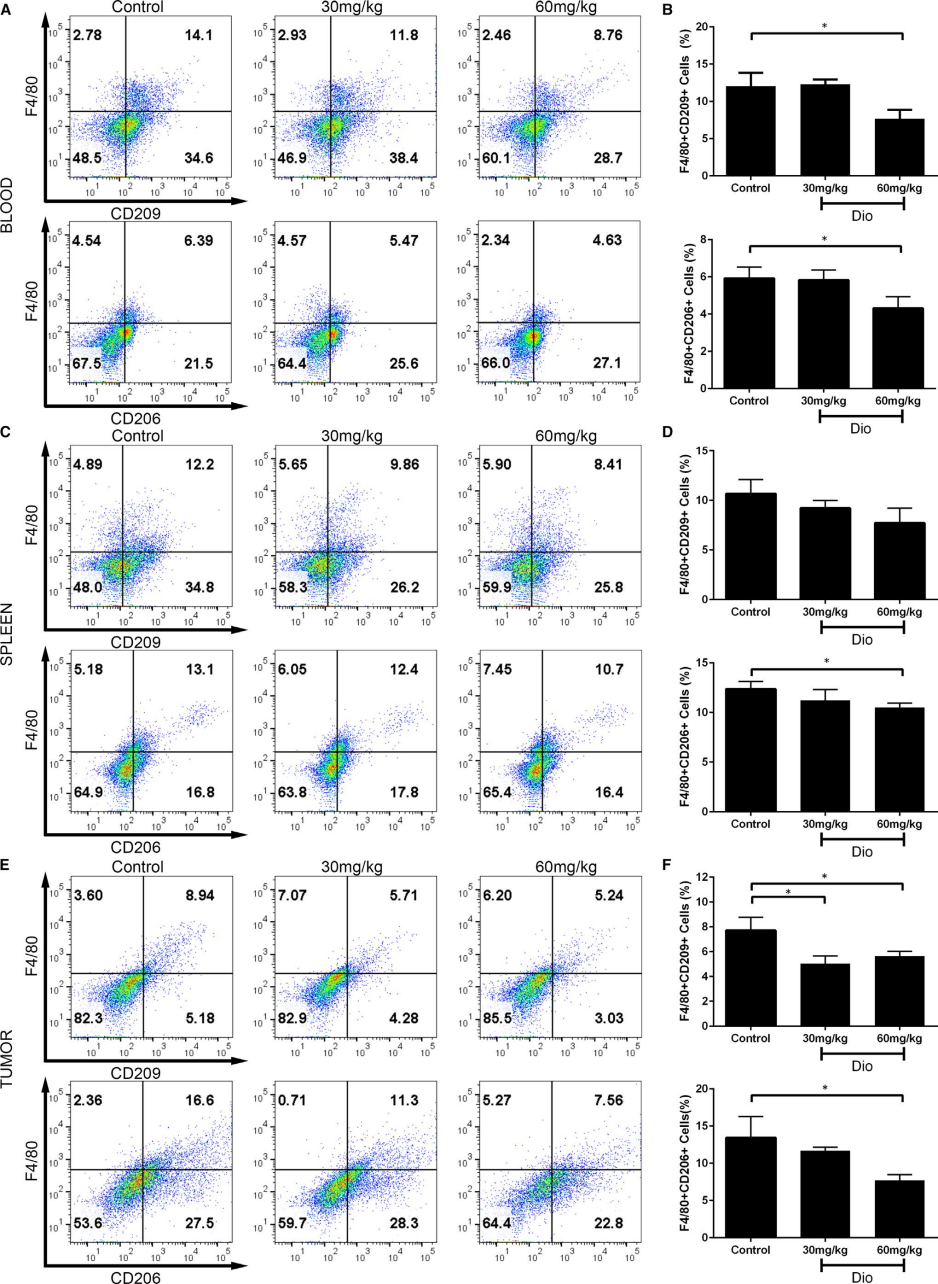
Dioscin restrained the augmentation of M2 macrophage populations in vivo. PBMCs, splenocytes and tumour tissue cells were collected from 3LL xenograft model. F4/80^+^CD206^+^ cells and F4/80^+^CD209^+^ cells were identified as M2 macrophages. The percentages of M2 macrophages in PBMCs (A‐B), splenocytes (C‐D), tumour tissue (E‐F) were shown. The data were representatives of three independent experiments and presented as the mean ± SD. *P < 0.05, **P < 0.01 and ***P < 0.001 vs control

To further verify the effects of dioscin in vivo, we constructed a subcutaneous lung tumour model. Peripheral blood mononuclear cells (PBMCs), splenocytes and tumour‐associated cells were collected and assessed by flow cytometry. M2 macrophages were marked as F4/80^+^CD206^+^ cells or F4/80^+^CD209^+^ cells. As shown in Figure 3, the percentage of F4/80^+^CD209^+^ macrophages in PBMCs was significantly decreased from 12.03 ± 1.79% for the control group to 7.70 ± 1.17% for the dioscin‐treated group (60 mg/kg) (*P* < 0.05). The proportion of F4/80^+^CD206^+^ cells with dioscin treatment (60 mg/kg) was decreased too (4.32 ± 0.62% vs 5.92 ± 0.60%, *P < *0.01). And in splenocytes, compared with the control group, 60 mg/kg dioscin treatment reduced the proportion of F4/80^+^CD206^+^ cells (10.50 ± 0.44% vs 12.37 ± 0.75%, *P < *0.05). The levels of F4/80^+^CD209^+^ cells were decreased from 10.69 ± 1.39% in the control group to 7.72 ± 1.48% in dioscin treatment (60 mg/kg) although with no significant statistical difference (Figure 3C,D). Dioscin treatment (30 mg/kg and 60 mg/kg) decreased the population of F4/80^+^CD209^+^ macrophages that infiltrated tumour cells (5.03 ± 0.62% and 5.66 ± 0.37% vs 7.71 ± 0.61%, respectively, both *P* < 0.05). Similarly, F4/80^+^CD206^+^ TAMs were dramatically decreased in 60 mg/kg dioscin treatment group (7.72 ± 0.76% vs 13.43 ± 2.84%, *P* < 0.05) (Figure 3E,F). In summary, dioscin inhibited the augmentation of M2 macrophage populations in mice with lung cancer.

### 3.4 Dioscin decreased the IL‐10 secretion of macrophages in vitro

**FIGURE 4:**
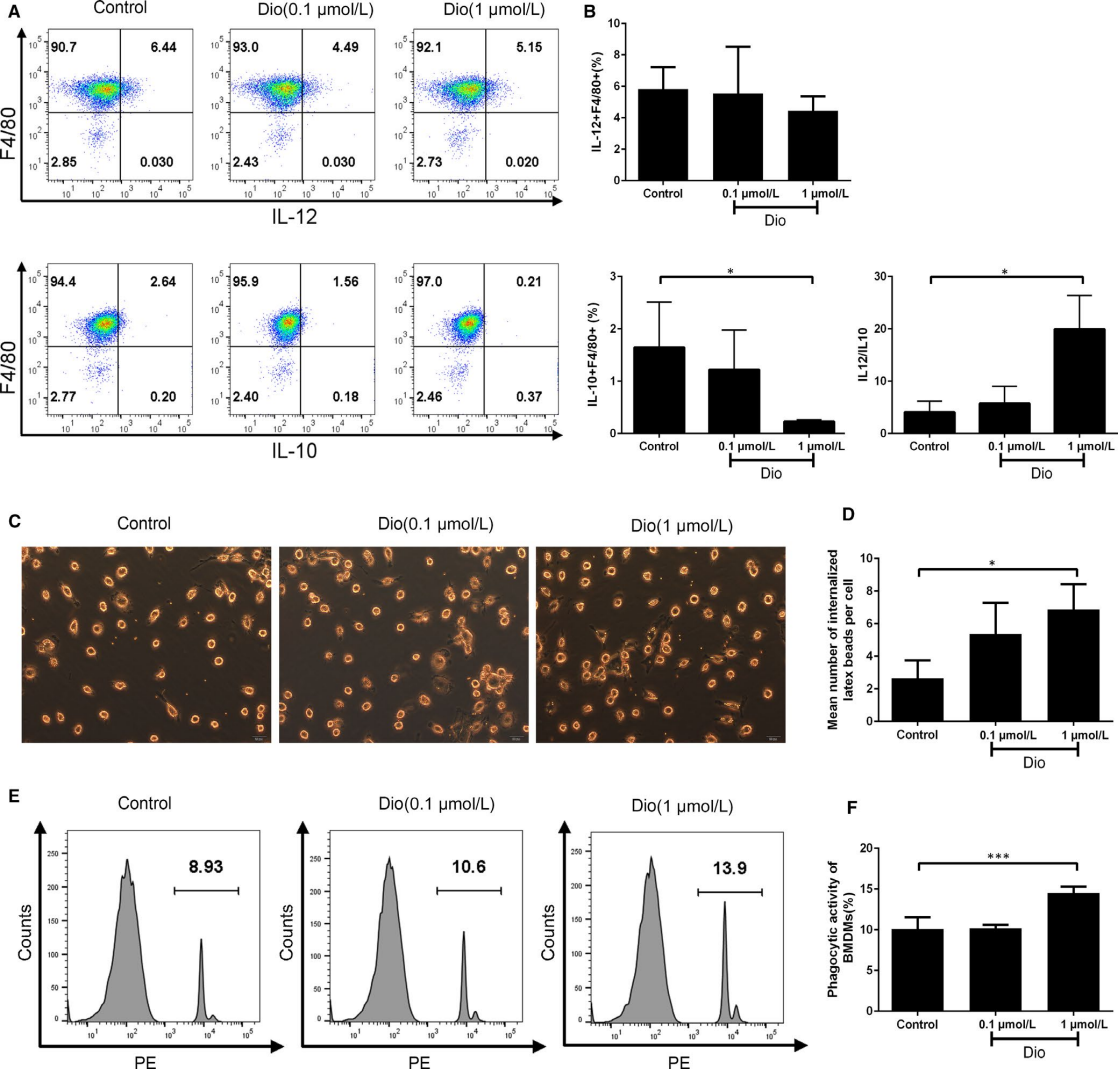
Dioscin affected cytokine secretion and enhanced the phagocytosis of BMDMs. BMDMs were treated with dioscin for 48 h. A‐B, The levels of IL‐10 and IL‐12 secreted by BMDMs were measured by flow cytometry. C‐D, The mean number of internalized latex beads per cell by microscopy. Scale bars: 20 μm. E‐F, the percentage of BMDMs internalizing latex beads was measured by flow cytometry. Data were the mean ± SD of triplicate independent experiments. *P < 0.05, **P < 0.01 and ***P < 0.001 vs control

To investigate the effect of dioscin on macrophage secretions, we used flow cytometry to analyse the intracellular cytokines. Compared with the control group, we found 1 μmol/L dioscin could significantly inhibit the level of IL‐10 secreted by BMDMs (0.23 ± 0.03% vs 1.65 ± 0.86%, *P* < 0.05), while could not influence IL‐12 secretion evidently. Interestingly, the ratio of IL‐12/IL‐10 was highly increased from 4.09 ± 2.08 in the control group to 19.96 ± 6.44 in 1 μmol/L dioscin group (*P* < 0.05) (Figure 4A,B).

### 3.5 Dioscin enhanced the phagocytosis of BMDMs

To study the effect of dioscin on phagocytosis, we used microscopy and flow cytometry to measure the amount of fluorescence‐labelled latex beads internalized by BMDMs, as described in Materials and Methods. As shown in Figure 4, 1 μmol/L dioscin treatment obviously raised the mean number of latex beads internalized by BMDMs (6.9 ± 1.6 vs 2.6 ± 1.1, *P* < 0.05). Compared with the control group, 1 μmol/L dioscin apparently increased the population of BMDMs internalizing latex beads (14.53 ± 0.78% vs 10.10 ± 1.44%, *P* < 0.001) (Figure 4E,F).

### 3.6 Dioscin down‐regulated STAT3 and JNK signalling pathways in Raw264.7 cells and BMDMs

**FIGURE 5:**
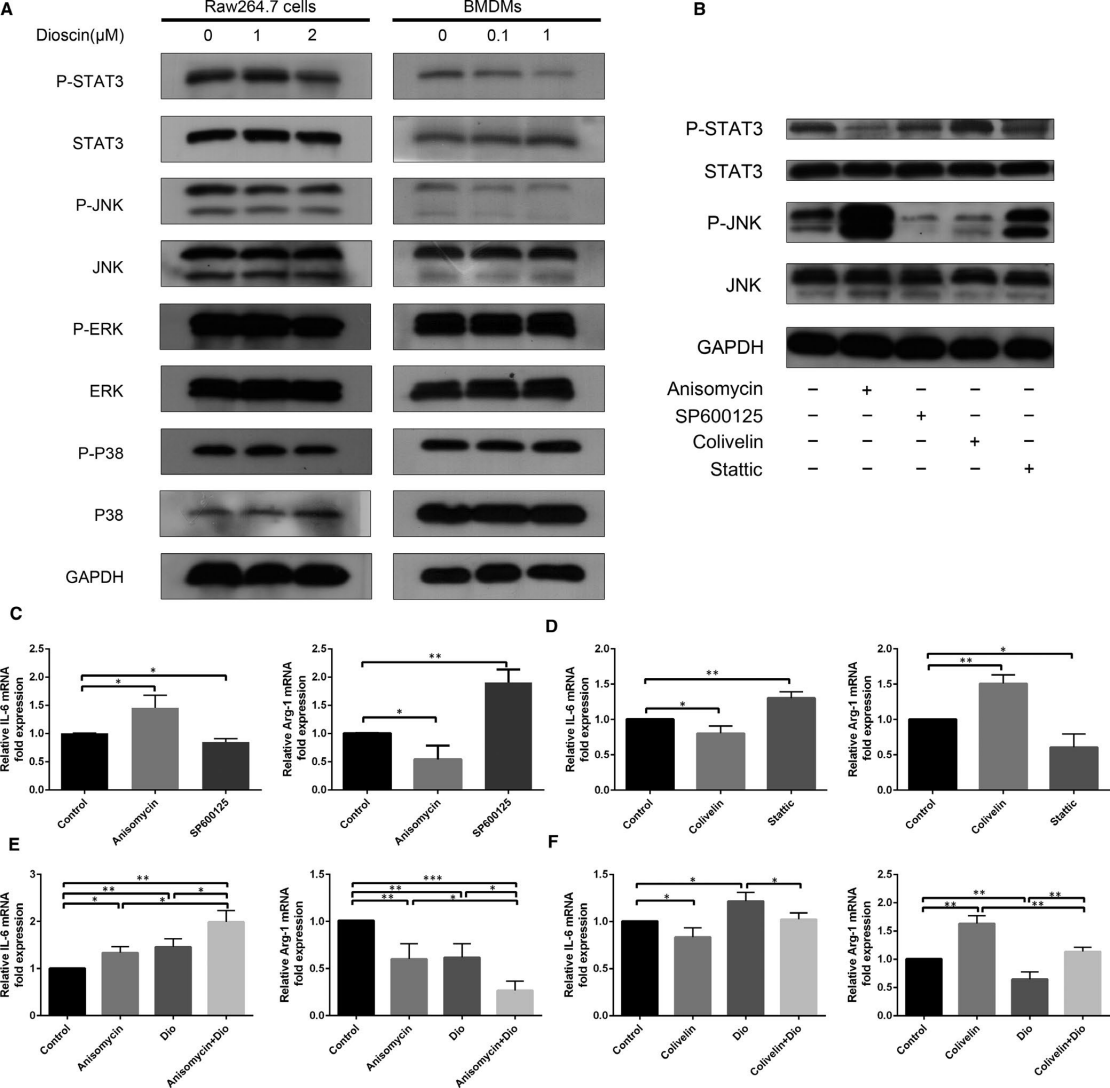
Dioscin down‐regulated STAT3 and JNK signalling pathways in macrophages. A, Raw264.7 cells and BMDMs were treated with dioscin for 48 h. The levels of p‐STAT3, p‐p38, p‐JNK and p‐ERK were analysed by Western blot. B, BMDMs were treated with JNK activator Anisomycin, JNK inhibitor SP600125, STAT3 activator Colivelin and STAT3 inhibitor Stattic. The levels of p‐STAT3 and p‐JNK were analysed by Western blot. C‐D, With activators and inhibitors treated, the relative expression of IL‐6 and Arg‐1 mRNA in BMDMs was analysed by qPCR. E‐F, BMDMs were pre‐incubated with Anisomycin and Colivelin and then treated with 1 μmol/L dioscin for 48 h. The relative expression of IL‐6 and Arg‐1 mRNA was analysed by qPCR. Data were presented as the mean ± SD of triplicate samples. *P < 0.05, **P < 0.01 and ***P < 0.001 vs control

We further evaluated the effect of dioscin on activation of STAT3 and the MAPK pathway in RAW264.7 cells and BMDMs. As shown in Figure 5A, compared with the control group, dioscin treatment (0.1 μmol/L and 1 μmol/L) significantly inhibited the phosphorylation of STAT3 and JNK in a low dose‐dependent manner. However, dioscin had no obvious effect on the p38 and ERK signalling pathway. In order to discover the role of STAT3 and JNK signalling pathways during dioscin‐mediated macrophage polarization, BMDMs were treated with different inhibitors and activators (Figure 5B). We found activating JNK and inhibiting STAT3 induced higher IL‐6 mRNA levels but lower Arg1 mRNA levels in BMDMs, while inhibiting JNK and activating STAT3 induced opposite results (Figure 5C,D). What's more, the effect of dioscin in macrophage polarization could be enhanced by Anisomycin and weakened by Colivelin (Figure 5E,F).

### 3.7 Dioscin‐mediated macrophage polarization suppressed the migration of 3LL cells

**FIGURE 6:**
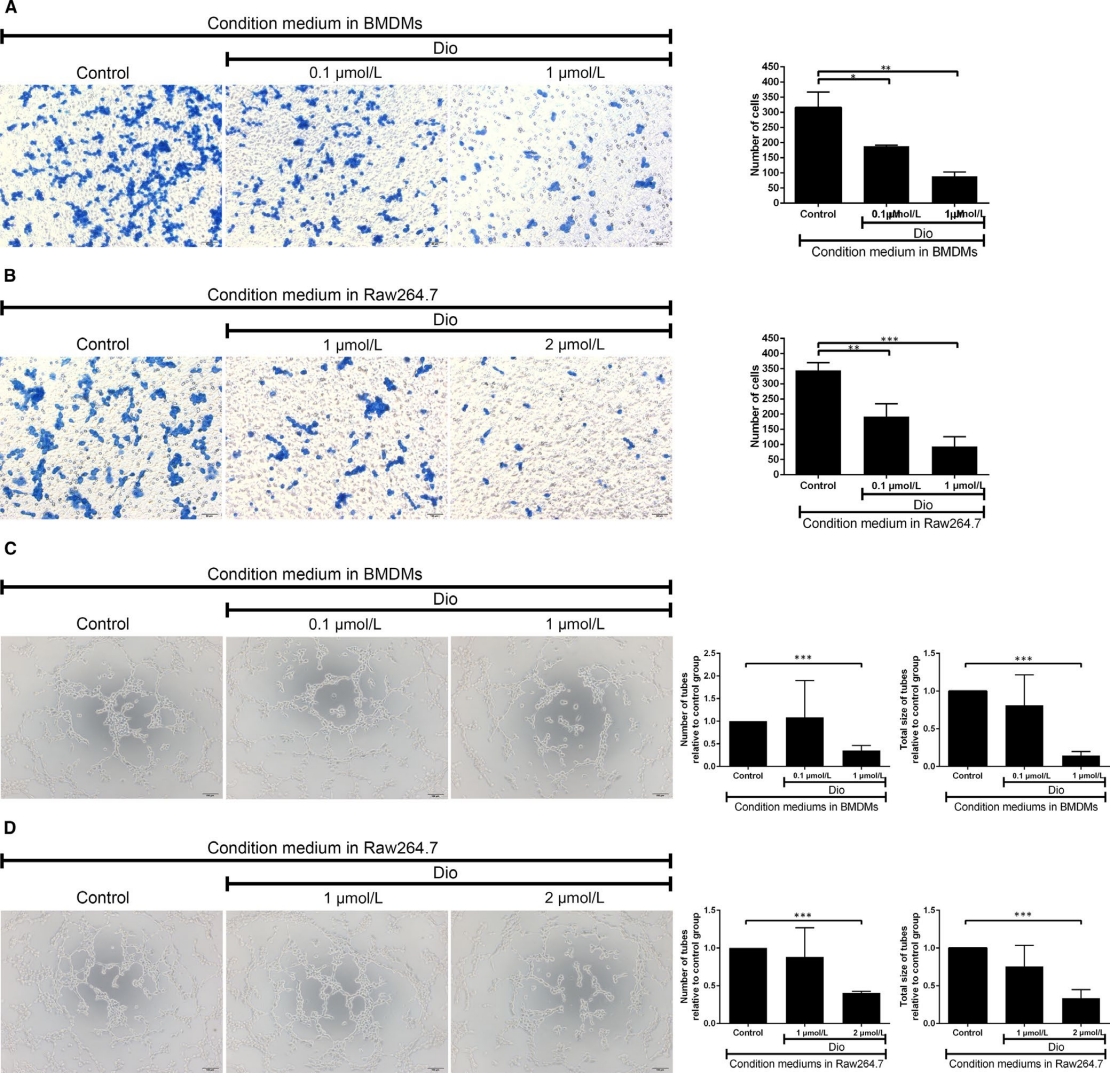
Dioscin‐mediated macrophage polarization suppressed the migration of 3LL cells and the tube‐formation capacity of HUVECs. Condition mediums were collected to treat 3LL cells and HUVECs. A‐B, Migration of 3LL cells was assessed by Transwell assays. Cells were counted by ImageJ. Scale bars: 50 μm. C‐D, The numbers and total size of tubes formatted by HUVEC were summed up by ImageJ. Scale bars: 100 μm. The data were presented as the mean ± SD of triplicate independent samples. *P < 0.05, **P < 0.01 and ***P < 0.001 represent a significant difference

Previous results exhibit dioscin could regulate macrophage polarization. However, whether dioscin could influence the metastasis of tumour by regulating macrophage polarization still unknown. We then collected condition medium from dioscin pre‐treated BMDMs and Raw264.7 cells to study the impact on 3LL cells’ migration. And we found dioscin (0.1 μmol/L and 1 μmol/L) pre‐treated condition medium suppressed the migration of 3LL cells in BMDMs (188 ± 3 and 88 ± 15 vs 316 ± 51, respectively, *P* < 0.05 and *P* < 0.01) (Figure 6A). While in Raw264.7 cells, after dioscin treatment (1 μmol/L and 2 μmol/L), the number of 3LL cells moved to the lower chamber was remarkably decreased (192 ± 41 and 93 ± 33 vs 344 ± 26, respectively, *P* < 0.01 and *P* < 0.001) (Figure 6B). Therefore, dioscin could regulate the polarization of macrophages to inhibit the migration of 3LL cells.

### 3.8 Dioscin‐mediated macrophage polarization inhibited the tube‐formation capacity of HUVECs

Similarly, we used the collected conditioned medium to treat the HUVECs. As shown in Figure 6C, 1 μmol/L dioscin pre‐treated condition medium from BMDMs could inhibited the tube formation. The number and total size of tubes relative to the control group was 0.35 ± 0.12 and 0.14 ± 0.05 (both *P *< 0.001). And in Raw264.7 cells, condition medium from 2 μmol/L dioscin treatment could apparently decreased the number and total size of tubes formed by HUVEC (0.40 ± 0.02 and 0.34 ± 0.11, relative to the control group, both *P* < 0.001) (Figure 6D). So, we believed dioscin inhibited the tube‐formation capacity of HUVECs by regulating macrophage polarization.

### 3.9 Dioscin inhibited the in vivo metastasis of 3LL cells through mediating macrophage polarization

**FIGURE 7:**
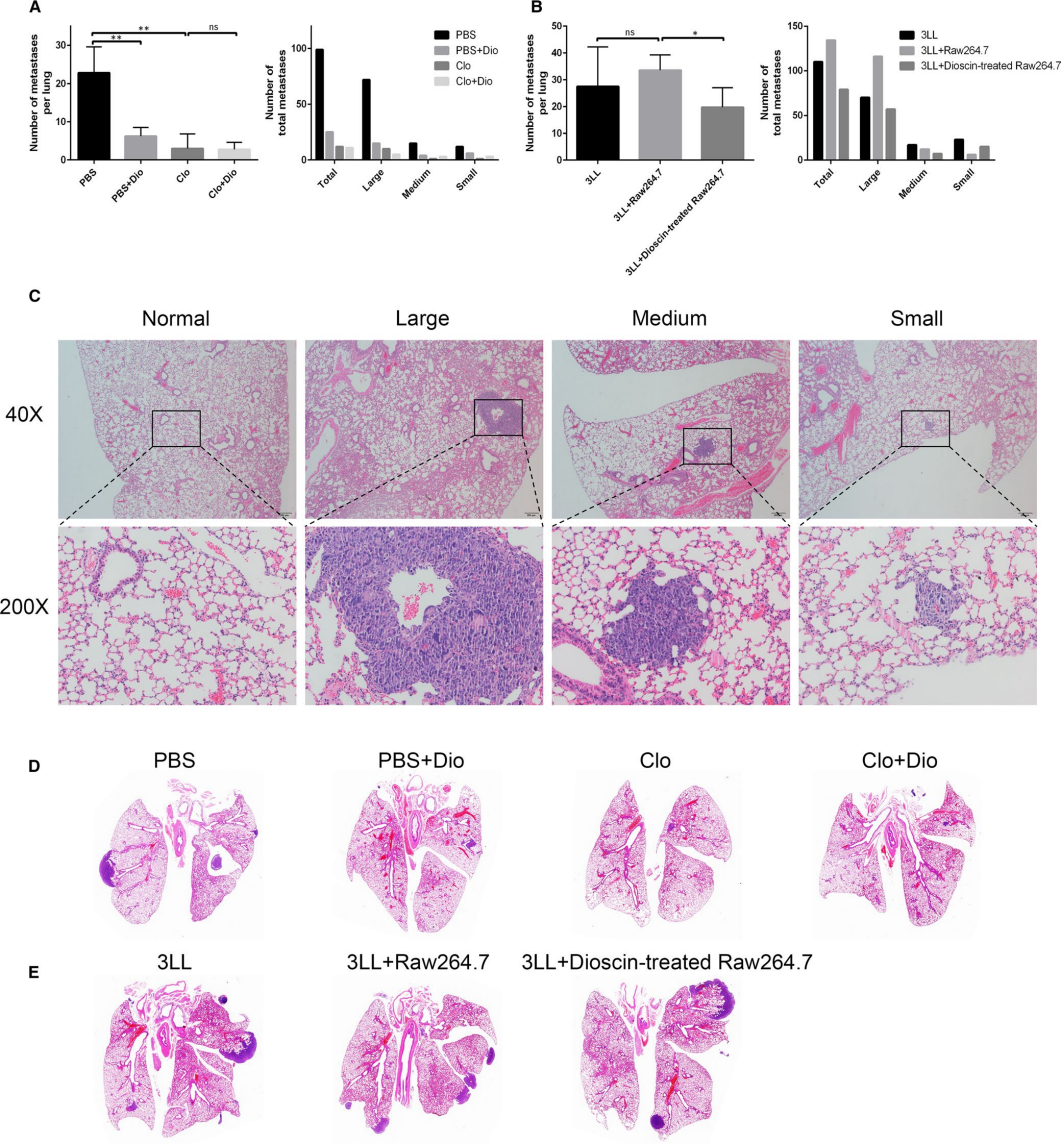
Dioscin‐mediated macrophage polarization suppressed the metastasis of 3LL cells in vivo. In lung metastases mouse model (A), C57BL/6 mice (n = 4) were injected intravenously with 3LL cells and treated with dioscin (60 mg/kg), clodronate liposome or both of them. In co‐inoculated lung metastasis mouse model (B), C57BL/6 mice (n = 4) were injected intravenously with 3LL cells alone or in combination with RAW264.7 cells not treated or exposed to 2 μmol/L dioscin for 48 h. After 21 d, the mice were sacrificed and their lungs were removed and histologically analysed for metastases. The number of metastases per lung and the number of small (diameter: <200 μm), medium‐sized (diameter: 200‐400 μm) and large (diameter: >400 μm) lung metastases were determined. C‐E, Representative images of the lungs. Scale bars: 200 μm. *P < 0.05, **P < 0.01 and ***P < 0.001 represent a significant difference

To verify the effect of dioscin on anti‐tumour metastasis, we constructed an intravenous injection lung metastasis mouse model. We also used clodronate liposomes to chemically eliminate macrophages to further confirm the anti‐tumour metastasis effect was related to macrophages. The total metastases per lung of dioscin‐treatment and clodronate liposome‐treatment were reduced dramatically (both *P* < 0.01) (Figure 7A). What's more, there were no difference between clodronate liposome combined with dioscin treatment and clodronate liposome treatment. This means after eliminating macrophages, the effect of dioscin on anti‐tumour metastasis was not obvious. Another co‐inoculated lung metastasis mouse model indicated dioscin‐treated Raw264.7 cells could inhibit the lung metastasis of 3LL cells (*P* < 0.05) (Figure 7B). Therefore, we demonstrated dioscin inhibited the lung metastasis of 3LL cells through regulating macrophage polarization in vivo.

## 4 DISSCUSION

In the recent years, dioscin has shown its powerful anti‐tumour activity. Many studies illustrate dioscin could induce autophagy, increase mitochondrial injury, enhance cell apoptosis, promote ROS accumulation and Ca^2+^ release to suppresses malignant activities of cancer cells.^24^, ^25^, ^26^, ^32^ And in human lung cancer cells, dioscin could inhibit proliferation and promote apoptosis by inducing DNA damage, cell cycle arrest and activating mitochondrial signalling pathways.^27^, ^28^ As tyrosine kinase inhibitors (TKIs) bring a new avenue for lung cancer patients, the drug resistance becomes an important issue in clinical work. Surprisingly, Wang et al demonstrate dioscin could overcome TKI resistance in EGFR‐mutated lung adenocarcinoma.^29^ To our knowledge, as not widely reported, the effect of dioscin on immunity remains obscure. Our study indicated dioscin could inhibit macrophage M2 polarization and enhance phagocytosis of macrophages with no significant side effects in vivo and in vitro. Furthermore, dioscin‐mediated macrophage polarization inhibited the metastasis of 3LL cells by suppressing migration and angiogenesis.

M1 and M2 are two activated phenotypes of macrophages exhibit vastly different functions. Classically activated macrophages (M1) release a series of pro‐inflammatory mediators and up‐regulated antigen presentation to defend against pathogen and eliminate malignant cells.^9^, ^15^, ^33^ On the contrary, alternatively activated macrophages (M2) participate in proliferation of cancer cells, angiogenesis, tumour invasion and metastasis to show a powerful tumour‐promoting role.^9^, ^13^ In many cancers (including lung cancers), TAMs were considered as M2 phenotype and promote tumour development in various ways.^9^, ^13^, ^14^, ^15^ In our study, M1 macrophages were marked as CD86, NOS2 and IL‐6 with high secretion of IL‐12 while M2 macrophages were marked as CD206, CD209 and Arg‐1 with high secretion of IL‐10. Our results confirmed dioscin could up‐regulated expression of M1 markers and down‐regulated expression of M2 markers in BMDMs and Raw264.7 cells. CD206 and CD209 are two classical markers to identify the M2 macrophages with confirmed specificity and reliability in lung cancers.^12^, ^33^, ^34^, ^35^, ^36^ Arg‐1 and NOS2 are two opposite markers to catabolize L‐arginine into different ingredients with different effects.^15^ It is worth noting that Arg‐1 may not be suitable for marking human M2 macrophages with not regulated by M2‐inducing cytokines in human.^33^ Besides our results even showed the phagocytosis of BMDMs was enhanced by dioscin. What's more, we constructed a subcutaneous lung tumour model to further verify the effects of dioscin in vivo. And we found dioscin inhibited the augmentation of M2 macrophage populations in PBMCs, splenocytes and tumour tissues, which was consistent with in vitro experiments. Based on above results, we demonstrated dioscin induced M2‐to‐M1 phenotype transition of macrophages and reappeared active defensive properties of macrophages. Some pharmacological molecules could re‐switch the polarization like dioscin and show significant enhancement of patients’ survival.^37^, ^38^, ^39^ Clinical researches also reveal the infiltration of M2‐like TAMs is associated with treatment failure and poor prognosis in different cancers.^2^, ^9^, ^12^ Thus, we have reasons to believe the potential anti‐tumour effect of dioscin by regulating macrophage polarization.

Increasing evidence illustrated signal transducer and activator of transcription (STAT) and mitogen‐activated protein kinase (MAPK) pathway are closely related to macrophage polarizations.^9^, ^40^, ^41^ Briefly, M1 polarization is induced by LPS and IFNγ with activation of STAT1 and NF‐κB.^9^, ^42^ IL‐4/IL‐13‐induced activation of STAT6 and IL‐10‐induced activation of STAT3 cause macrophage M2 polarization.^5^, ^41^, ^42^ Hua et al found the overexpression of STAT3 in malignant cells resulted in the activation of STAT3 of surrounding stroma cells, which finally caused TAMs M2 polarization.^43^ By activating STAT3, TAMs and small‐cell lung cancers could jointly promote tumour progression.^44^ What's more, the activation of STAT3 up‐regulated angiogenic, metastatic and pro‐proliferation relevant gene expression.^43^ MAPK pathway, including ERK, p38 and JNK, is disputable in macrophage polarization. Previous studies confirm the activation of ERK and p38 induces M2 macrophage polarization while JNK for M1 polarization.^12^, ^41^, ^45^, ^46^ Oppositely, others believe activated‐JNK leads to M2 polarization.^16^ Moreover, IL‐4, a M2‐inducing mediator, could activate JNK and promote proliferation of cancer cells simultaneously.^47^ Our study showed dioscin, may as a potential IL‐10 inhibitor, down‐regulated the expression of activated STAT3 based on phenotype transform. And STAT3 inhibitor Stattic induced macrophages to M1 polarization, and STAT3 activator Colivelin induced macrophages to M2 polarization. What's more, the effect of dioscin in macrophage polarization could be reversed by Colivelin. In accord with our findings, other study has shown anti‐IL‐10 receptor antibodies and STAT3 inhibitors induced macrophage phenotype from M2 to M1 switch.^2^ We also found the expression of activated JNK was down‐regulated by dioscin with no significant change in p38 and ERK. A recent study demonstrated Arg‐1 expression varies as the JNK expression changes simultaneously in IL‐4–induced M2 macrophage polarization.^16^ However, our results illustrate JNK activator Anisomycin down‐regulated relative expression of Arg‐1 mRNA and enhanced the effect of dioscin.

Tumour metastasis is a complex invasion‐metastasis cascade requiring angiogenesis, damage of the basement membrane and remodelling of the extracellular matrix for malignant cell migration, invasion and extravasation.^48^, ^49^ It is known that M2‐like TAMs induce immunosuppressive tumour microenvironment to promote dissemination of malignant cells in the early stage of tumour metastasis.^9^, ^19^, ^48^ A notably study illustrated the augmentation of M2‐like TAMs in lung adenocarcinoma was associated with tumour metastasis.^12^ This study confirmed condition medium from dioscin pre‐treated macrophages (BMDMs and Raw264.7 cells) inhibited the migration of 3LL cells and angiogenesis of HUVECs. Kou et al also elucidated the similar results in melanoma cells.^10^ What's more, M2 macrophages could promote human lung cancer cell migration and up‐regulate the expression of VEGF and MMPs for angiogenesis and invasion.^34^ Based on the effect of dioscin on macrophage polarization, we believe dioscin influenced relevant mediators released by macrophages to inhibit angiogenesis and cancer cell migration. Moreover, our lung metastasis model experiment demonstrated dioscin suppressed the lung metastasis of 3LL cells in vivo, which was correlated with macrophages. And dioscin‐treated macrophages inhibited the lung metastasis of 3LL cells. Thus, we concluded dioscin could inhibit the lung metastasis of 3LL cells through regulating macrophage polarization. In conclusion, our study demonstrates a novel anti‐tumour effect of dioscin by inhibiting macrophage M2 polarization via JNK and STAT3 pathways in lung cancer.

## CONFLICT OF INTEREST

The authors confirm that there are no conflicts of interest.

## AUTHOR CONTRIBUTION

**Luyun Cui:** Conceptualization (equal); Formal analysis (equal); Methodology (equal); Project administration (lead); Writing‐original draft (equal); Writing‐review & editing (equal). **Guangdie Yang:** Conceptualization (supporting); Formal analysis (equal); Methodology (equal); Project administration (equal); Writing‐original draft (equal); Writing‐review & editing (equal). **Jiani Ye:** Project administration (equal); Writing‐review & editing (supporting). **Yinan Yao:** Funding acquisition (supporting); Project administration (supporting); Writing‐review & editing (supporting). **Guohua Lu:** Project administration (supporting); Writing‐review & editing (supporting). **Junjun Chen:** Formal analysis (supporting); Funding acquisition (supporting). **Liangjie Fang:** Formal analysis (supporting); Funding acquisition (supporting). **Shan Lu:** Formal analysis (supporting); Funding acquisition (supporting). **Jianying Zhou:** Conceptualization (equal); Funding acquisition (lead); Methodology (equal); Writing‐review & editing (lead).

## Data Availability

The data that support the findings of this study are available from the corresponding author upon reasonable request.
